# Preparation of Paper-Based Fluorescent Sensors and Their Application for the Detection of Cu^2+^ in Water

**DOI:** 10.3390/ma17163920

**Published:** 2024-08-07

**Authors:** Yue Ma, Hui Li, Yufeng Li, Dong Wei

**Affiliations:** 1 College of Agriculture and Forestry, Hebei North University, Zhangjiakou 075000, China; hbgcmy@163.com (Y.M.); lihui924@126.com (H.L.); 2 Hebei Key Laboratory of Quality and Safety Analysis of Agricultural Products and Food, Hebei North University, Zhangjiakou 075000, China; 3 Key Laboratory of Quality and Safety of Zhangjiakou Special Agricultural Products, Hebei North University, Zhangjiakou 075000, China

**Keywords:** paper, polyethyleneimine, fluorescence, detection, Cu^2+^

## Abstract

Excessive copper (Cu^2+^) causes adverse effects on human health and the ecological environment. Traditional methods for detecting Cu^2+^ have drawbacks such as high detection costs, complex operating conditions, and being time consuming. Therefore, there is an urgent need to develop simple detection methods to better meet specific health and environment quality needs. In this work, a paper-based fluorescence sensor was prepared (herein referred to as the as-prepared method) by immersing filter paper in aqueous polyethyleneimine (PEI) solution, and its potential use in Cu^2+^ detection was investigated. The results showed that the as-prepared paper samples, with fluorescence properties obtained by aggregation-induced luminescence of PEI, have selective recognition of Cu^2+^ based on the internal filtration effect, and the lowest detection limit is 0.03 μM. In addition, the relative error of this method is in the range of 1.80~2.23%, which is relatively comparable to the national standard method (0.63~630 μM), demonstrating high accuracy. Therefore, paper-based sensors with a simple preparation method have potential applications in the detection of Cu^2+^ in water.

## 1. Introduction

Copper (Cu^2+^) is a trace element that is essential for the human body and plays critical roles in some physiological and pathological processes such as bone formation and cell respiration. A deficiency of Cu^2+^ in the human body can cause diseases such as anemia, vision loss, and bone loss [1,2]. However, an excess of Cu^2+^ in the body may destabilize the intracellular environment, thus inducing or aggravating liver- and kidney-related diseases, as well as Alzheimer’s disease, Wilson’s disease, Menke’s disease, and Parkinson’s disease, which seriously harm people’s health [3,4,5]. Recently, the pollution of water quality by Cu^2+^ has become more and more serious. So, the Chinese National Research Council set the recommended daily intake of Cu^2+^ at 2.0 to 3.0 mg for adults, 1.5 to 2.5 mg for children, and 0.5 to 1.0 mg for infants [6]. In addition, the maximum allowable amount of Cu^2+^ in drinking water, as set by the World Health Organization, is 1 mg L^−1^ (about 15.75 μM) [7]. Therefore, the monitoring of Cu^2+^ in water is particularly important for human health and environmental protection.

At present, the main methods for detecting Cu^2+^ in water include atomic absorption spectrometry [8,9], atomic fluorescence spectrometry [10,11], inductively coupled plasma mass spectrometry [12], and electrochemical analysis [13,14]. Although these methods can quickly and accurately detect the content of Cu^2+^, they have disadvantages such as complicated operation processes, expensive equipment acquisition, and high requirements for the operating environment. In addition, compared with the above methods, optical methods such as fluorescence and ultraviolet–visible (UV–vis) spectrophotometry are also widely used for the detection of Cu^2+^ due to their simple operation, and low cost and instrument requirement [15,16]. Moreover, fluorescence spectrophotometry has some advantages such as a higher sensitivity, a stronger selectivity, and a lower detection limit compared with UV–vis spectrophotometry [17,18,19,20,21]. However, there is still an urgent need to develop detection methods that are easy to operate, portable, and do not require auxiliary equipment to meet the needs of certain special fields.

Paper, mainly composed of cellulose, has the characteristics of flexibility, being lightweight, and having a low cost, and has been widely used in packaging, decoration, and other fields [22,23,24]. In addition, due to its abundant pore structure and portability, paper as a carrier has been widely used in the field of the detection of heavy metal ions in recent years. For instance, Shi et al. synthesized a fluorescent sensor with a pyrazine unit based on the excited-state intramolecular proton transfer process, which could not only be used to measure Cu^2+^ in real water samples accurately, but could also be made into paper strips for the determination of Cu^2+^ [25]. Sadollahkhani et al. prepared a colorimetric disposable paper coated with ZnO@ZnS core–shell nanoparticles for the detection of Cu^2+^ with a visual detection limit as low as 15 µM [26]. Further, Zhang et al. developed a detecting system consisting of AuNCs@ZIF-8-modified paper and a smartphone platform for the visual detection of Cu^2+^ in agricultural samples [27]. All of the above studies indicate the potential application of paper-based sensors in the detection of heavy metal ions; however, the complex preparation procedures still limit the application range of paper-based fluorescence sensors. Polyethyleneimine (PEI) possesses a high density of amino groups, which can easily be adsorbed on the pristine paper through electrostatic interactions, hydrogen bonding, and van der Waals forces. Moreover, the inherent fluorescence characteristics of PEI have been reported in a few studies. Unfortunately, the fluorescence characteristics of paper-based sensors doped with PEI have been rarely investigated.

In this study, PEI was used to modify the surface of filter paper to prepare fluorescent paper-based materials. Fourier transform infrared spectroscopy (FTIR), scanning electron microscopy (SEM), atomic force microscopy (AFM), and X-ray photoelectron spectroscopy (XPS) were used to explore the interaction mechanism between PEI and pristine paper. The visual detection of paper samples for Cu^2+^ was evaluated with Image J 1.53 software. The research results provide a new idea for the simple preparation and application of new paper-based sensors.

## 2. Materials and Methods

### 2.1. Materials

Qualitative filter paper was obtained from Xinhua filter paper Co., Ltd. (Hangzhou, China). PEI (Mw = 600; 1800; 10,000; 25,000; 70,000 g mol^−1^) was gained from Macklin Biochemical Co., Ltd. (Shanghai, China). Potassium sulfate (K_2_SO_4_), copper sulfate (CuSO_4_), aluminum sulfate (Al_2_(SO_4_)_3_), sodium chloride (NaCl), mercury chloride (HgCl_2_), ferric chloride (FeCl_3_), zinc acetate ((CH_3_COO)_2_Zn), lead acetate ((CH_3_COO)_2_Pb), silver nitrate (AgNO_3_), sodium nitrate (NaNO_3_), sodium sulfate (Na_2_SO_4_), and sodium carbonate (Na_2_CO_3_) were of analytical grade and were purchased from Kemiou fine chemicals Co., Ltd. (Tianjin, China). Distilled water was used throughout the experiment.

### 2.2. Preparation of Paper Samples

PEI was deposited on the filter paper as a substrate using an immersion process. Firstly, the given substrate was dipped into a PEI aqueous solution for a given time. Secondly, the above substrate was rinsed with deionized water three times. Finally, the above substrate was dried at room temperature for 24 h and was sealed in a self-sealing bag.

### 2.3. Characterization of Paper Samples

FTIR was carried out using FTIR spectrometers (Perkin-Elmer, Shelton, CT, USA) for the characterization of chemical structures. The morphology of the paper samples was performed using an SS-35 SEM (Shimadzu, Kyoto, Japan) under a 5 kV acceleration voltage. AFM images of the paper samples were recorded using an AFM (JEM-2100, JEOL, Tokyo, Japan). XPS was carried out using an XPS (Thermo-Fisher, Waltham, MA, USA) for the analysis of elemental composition and chemical bond changes on the surface of the materials. The fluorescence spectrum was analyzed using an F-7000 fluorescence spectrophotometer (Perkin-Elmer, Shelton, CT, USA). The UV–vis spectrum was obtained using an F-1900 UV–vis spectrophotometer (Perkin-Elmer, Shelton, CT, USA).

### 2.4. Determination of Adsorption Capacity of PEI

The absorbance of PEI was measured using UV–vis spectrophotometry. Its content was obtained from the standard curve (y = 7.8776x + 0.3477, R^2^ = 0.9991, where x is mass concentration, and y is absorbance) and then the adsorption capacity of PEI was calculated.

### 2.5. Determination of Fluorescence Intensity of Paper Samples

The fluorescence intensity of the paper samples was measured using a fluorescence spectrometer. Before the test, the paper samples (1.5 cm × 3 cm) were fixed to the solid support with double-sided tape; the excitation slit was 5 nm, the emission slit was 5 nm, the gain was 2, and the voltage was 700 V.

### 2.6. The Effect of Common Ions on Fluorescence Intensity of Paper Samples

In order to explore the effect of Cu^2+^, Zn^2+^, Fe^3+^, K^+^, Na^+^, Hg^2+^, Al^3+^, Pb^2+^, Ag^+^, Cl^−^, SO_4_^2−^, CO_3_^2−^, and NO_3_^−^ on the fluorescence intensity of paper samples, the as-prepared paper sample (1.5 cm × 3.0 cm) was immersed in an aqueous solution, containing the corresponding ion (200 μM, 20 mL) and was oscillated for 30 min. Then, it was taken out and dried at room temperature. Finally, the dried paper sample was observed under 365 nm with an ultraviolet analyzer and photos were taken using a smartphone.

### 2.7. Quantitative Analysis of Color of Paper Samples

In order to analyze color, photographs of the paper samples were taken with the help of a smartphone (Huawei, Shenzhen, China, 600 dpi resolution). The color intensity was determined using Image J software, which is a free image processing program. This software accords the RGB (red, green, and blue) color of an image and then outputs, quantitatively, the intensity value of the testing area. The Intensity reported is the result subtracted from the blank. The lowest detection limit was calculated by multiplying the standard deviation of the blank by three and dividing it by the slope of the calibration curve.

### 2.8. Analysis of Real Water

To verify the application potential of the as-prepared paper samples, the Cu^2+^ content in tap water and lake water was measured. The tap water and lake water samples were taken from Hebei North University (Zhangjiakou, east longitude 114°52′67″, northern latitude 40°48′37″) and Xuan hua Artificial Lake (Zhangjiakou, east longitude 114°52′67″, northern latitude 40°48′37″), respectively. Before testing, the water sample was filtered with a 0.45 μm filter membrane. Then, the Cu^2+^ content of the water samples was determined according to the above method established in this study, and was compared with the Bis-cyclohexanone Oxalydihydrazone (BCO) spectrometric method where Cu^2+^ reacts with BCO in sodium borate buffer solution at pH 9.0~9.5 to form a blue complex, which can then be used for the determination of Cu^2+^ content at 600 nm using a spectrophotometer.

## 3. Results

### 3.1. Adsorption of PEI on Pristine Paper

#### 3.1.1. The Effect of Molecular Weight on Fluorescence Intensity of Paper Samples

**Figure 1:**
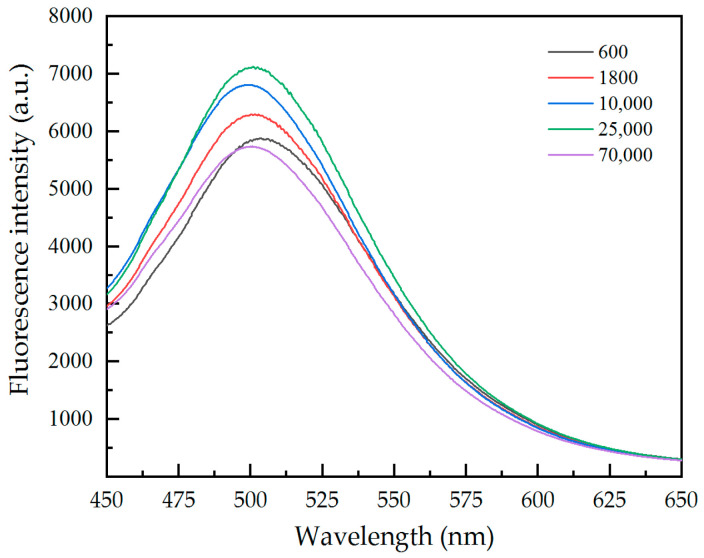
The effect of the molecular weight of PEI on the fluorescence intensity of paper samples.

It has been shown that PEI molecular chains can produce aggregation-induced emission when their structure is limited. In this experiment, the combination of PEI and pristine paper limited its structure. At the same mass concentration, PEI molecules with a low molecular weight have fewer binding sites with the pristine paper, less molecular chain restriction, and a lower fluorescence intensity. However, when the molecular weight of PEI is too large, the movement of its molecular chain is weakened, which will also limit its binding site with the pristine paper. The results show that molecular weight of PEI has a significant effect on the fluorescence intensity of the paper samples. When the molecular weight is 25,000, the fluorescence intensity of the paper samples is at its highest (Figure 1).

#### 3.1.2. The Effect of Mass Concentration on Fluorescence Intensity of Paper Samples

**Figure 2:**
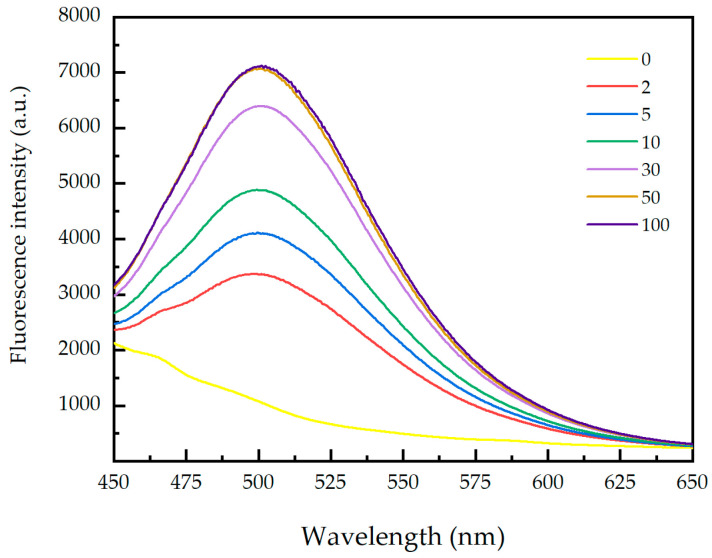
The effect of mass concentration on the fluorescence intensity of paper samples; the unit of the data is g L^−1^.

As can be seen in Figure 2, the concentration of PEI has a significant impact on the adsorption capacity to pristine paper. In general, the higher the concentration of the adsorbent, the greater its adsorption capacity. This is because an increase in adsorbent concentration can increase the collision probability between adsorbent molecules and adsorbed molecules, increasing the possibility of adsorption, thus increasing the adsorption capacity. However, when the concentration of PEI reaches a certain value, the space between the adsorbent molecules and PEI is limited. As the concentration continues to increase, it is difficult for PEI to find empty positions for adsorption. At this point, the adsorption capacity no longer increases. Therefore, a PEI solution with a mass concentration of 50 g L^−1^ can be used to prepare the paper-based fluorescence sensor.

#### 3.1.3. The Effect of Dipping Time on the Adsorption Capacity of PEI

**Figure 3:**
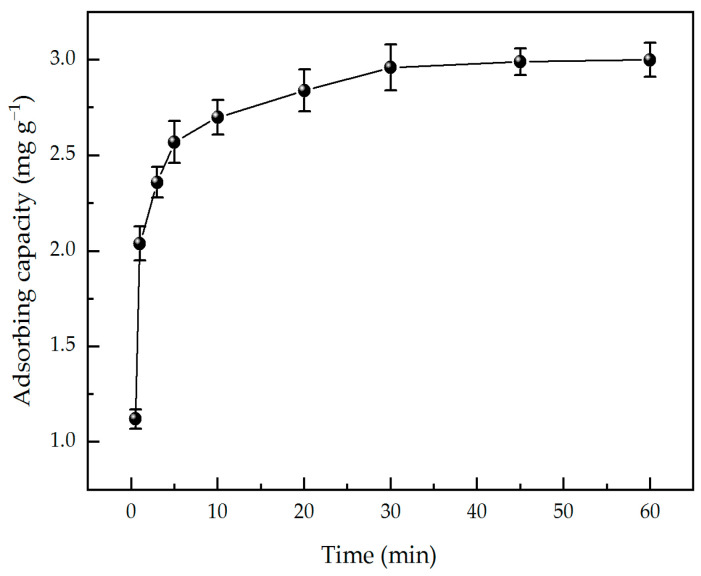
The effect of dipping time on the adsorption capacity of PEI.

As can be seen from Figure 3, adsorption time has a significant effect on adsorption capacity. At the initial stage, with the increase in time, the adsorption capacity of PEI increased rapidly. This is because at this stage, there are more active sites on the surface of the pristine paper, which can quickly adsorb PEI in the solution. With the further extension of time, the rate of increase in the adsorption capacity gradually slowed down, finally reaching an equilibrium state. This is because the active site on the surface of the pristine paper is gradually occupied and reaches a saturated state. At this time, even if the adsorption time is prolonged, the adsorption capacity is no longer obvious. The results show that the paper samples can be prepared by immersing the pristine paper in aqueous PEI solution for 30 min.

### 3.2. Characterization of Paper Samples

#### 3.2.1. FTIR Analysis of Paper Samples

**Figure 4:**
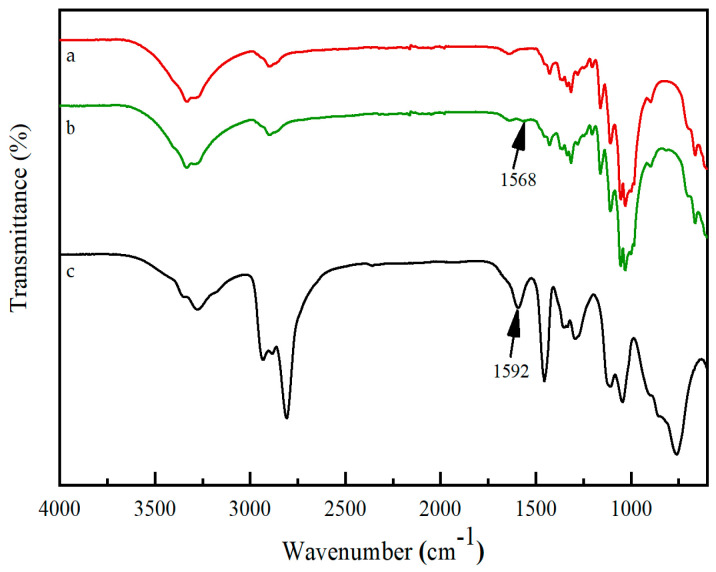
FTIR spectra of paper samples before (**a**) and after (**b**) adsorption of PEI and PEI (**c**).

FTIR spectra are commonly used to analyze the chemical structure of substances. As shown in Figure 4c, the characteristic peak at 1592 cm^−1^ is attributed to the asymmetric bending of the primary amines (–NH_2_) of PEI [28]. Compared with Figure 4, which is the FTIR spectra of the pristine paper, there is a new characteristic peak at 1568 cm^−1^ that is related to PEI, which undergoes a blue shift after the adsorption of PEI (Figure 4b). As is well known, the interactions between the functional groups of substances can affect the intensity and position of their characteristic peaks. The results show that PEI is successfully deposited on the pristine paper through various weak interactions.

#### 3.2.2. SEM Analysis of Paper Samples

**Figure 5:**
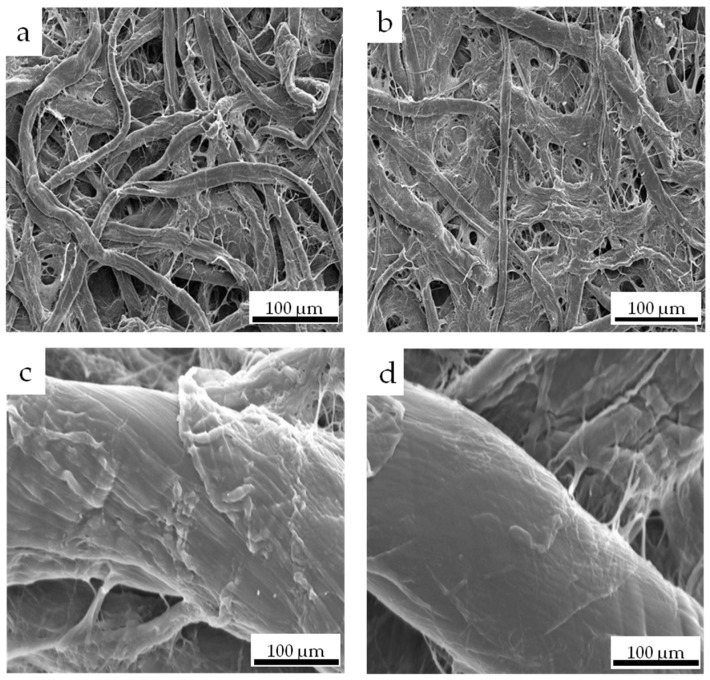
SEM images of the paper samples before (**a**,**c**) and after (**b**,**d**) the adsorption of PEI.

As can be seen from Figure 5a, the pristine paper is composed of fibers arranged in an unordered manner, and is filled with a large number of pores. However, the number of pores on the surface of the pristine paper after the adsorption of PEI is significantly reduced (Figure 5b). This is mainly because PEI has several hydrophilic amine groups, which can interact with functional groups such as hydroxyl groups and carboxylate through hydrogen bonding, electrostatic interactions, and van der Waals forces, thereby filling the pores on the surface of the original paper. In addition, the fibers of the pristine paper have several different textures and are relatively rough (Figure 5c), while the fibers after the adsorption of PEI become smoother and more delicate (Figure 5d). These results further confirm the successful deposition of PEI on the surface of the pristine paper.

#### 3.2.3. AFM Analysis of Paper Samples

**Figure 6:**
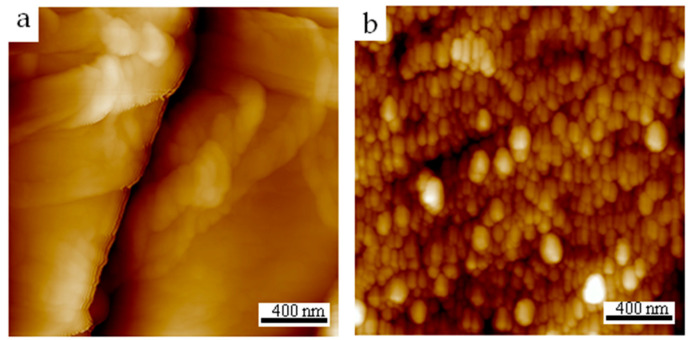
AFM images of the paper samples before (**a**) and after (**b**) the adsorption of PEI.

The ultrastructure of the paper surface was observed using AFM. As shown in Figure 6a, the pristine paper consists of long-range ordered fibers. After the adsorption of PEI, granular substances with a size of about 50~100 nm were deposited on the surface of the fiber, indicating that the immersion process achieved successful PEI adsorption (Figure 6b). The formation of granular material indicates that the molecular chains of PEI are bound, and this structural feature contributes to the aggregation-induced luminescence of PEI on the paper samples. In addition, the roughness of the paper samples decreased from 98.6 to 15.9, which may be related to the nanoparticles filled between the fibers after the adsorption of PEI.

### 3.3. Analysis of Fluorescence Properties of Paper Samples

**Figure 7:**
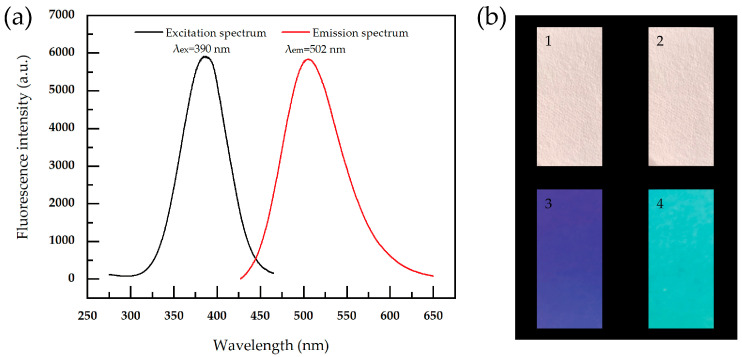
(**a**) Fluorescence excitation and emission spectra of the paper samples after the adsorption of PEI. (**b**) The images of the paper samples before (1,3) and after (2,4) the adsorption of PEI; images 1 and 2 were under visible light, while images 3 and 4 were under 365 nm UV light.

Figure 7a shows the excitation and emission spectra of the paper samples after the adsorption of PEI, with maximum excitation and emission wavelengths of 390 nm and 502 nm, respectively, and a large Stokes shift, indicating that the as-prepared paper samples belong to a fluorescent material. Figure 7b shows the optical images of the paper samples before and after the adsorption of PEI under visible light and 365 nm ultraviolet light. There is no obvious difference in the color of the two types of paper samples under visible light, but they show different luminous characteristics after ultraviolet light excitation. Under ultraviolet excitation, the dark blue fluorescence of the pristine paper may be related to the presence of fluorescent dyes. The cyan fluorescence emitted by the paper samples may be related to PEI molecules with restricted molecular chain structures.

### 3.4. Application of Paper Samples for Detection of Cu^2+^

#### 3.4.1. The Effect of Common Ions on the Fluorescence Intensity of Paper Samples

**Figure 8:**
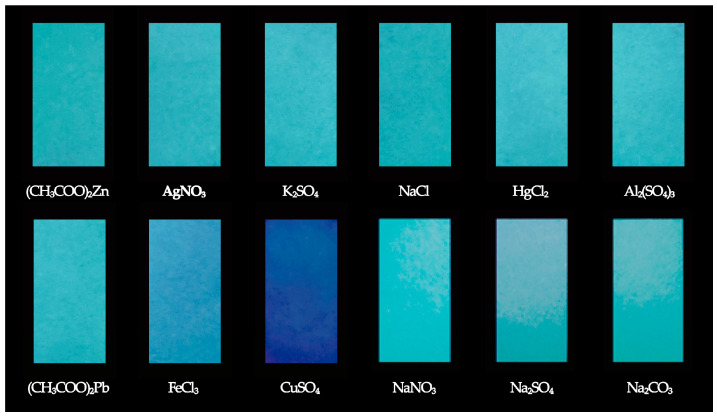
Fluorescence images of the as-prepared paper samples treated with common ions (200 μM) under 365 nm UV light.

The effect of common ions on the fluorescence intensity of the paper samples was evaluated. As can be seen from Figure 8, only paper samples after the adsorption of Cu^2+^ showed obvious color changes, and the effect of other common anions and cations on the color of the paper samples was negligible under 365 nm ultraviolet excitation. Therefore, the as-prepared paper samples present a high anti-interference performance and can selectively detect Cu^2+^, which can be used as a paper-based fluorescence sensor to detect Cu^2+^ in water samples.

#### 3.4.2. Detection of Paper Samples for Cu^2+^

**Figure 9:**
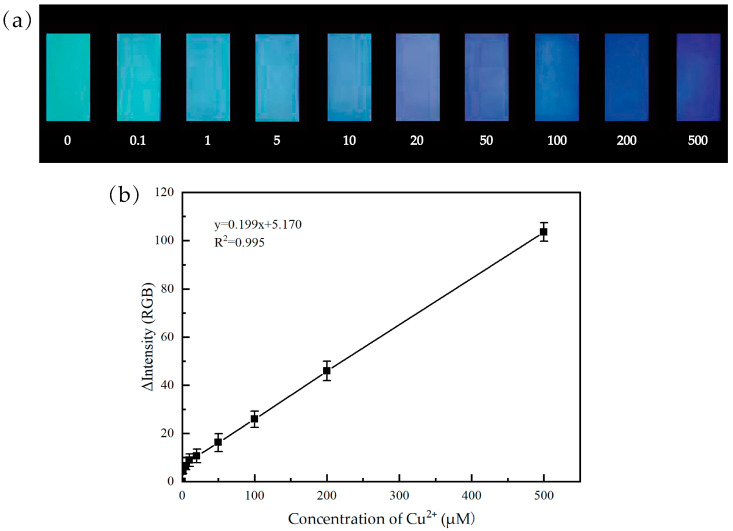
(**a**) Color change in the paper-based fluorescence sensors treated with different concentrations of Cu^2+^ under 365 nm UV light; the unit of the data is μM. (**b**) Calibration curve for the paper-based fluorescence sensors measured according to color intensity.

The response of the as-prepared paper samples to different concentrations of Cu^2+^ was further examined. As can be seen from Figure 9a, the color of paper-based fluorescence sensors gradually turns blue with the increase in Cu^2+^ concentration. Therefore, the paper-based fluorescence sensor can be used for the visual semi-quantitative detection of Cu^2+^. In order to further improve the accuracy, the paper samples adsorbed with different concentrations of Cu^2+^ were excited using UV, before being photographed, analyzed, and processed using Image J software. Notably, when the concentration of Cu^2+^ ranges from 1 μM to 500 μM, the change in color of the paper-based fluorescence sensors presents a good linear relationship with the concentration of Cu^2+^ (Figure 9b). The obtained linear regression equation is y = 0.199x + 5.170; the correlation coefficient R^2^ = 0.995; and the minimum detection limit is 0.03 μM, which is far below the World Health Organization’s maximum allowable Cu^2+^ in drinking water of about 15.75 μM. Therefore, the as-prepared paper-based fluorescence sensors can also effectively achieve the fluorescence quantitative detection of Cu^2+^ in water.

#### 3.4.3. Detection Mechanism of Paper Samples for Cu^2+^

**Figure 10:**
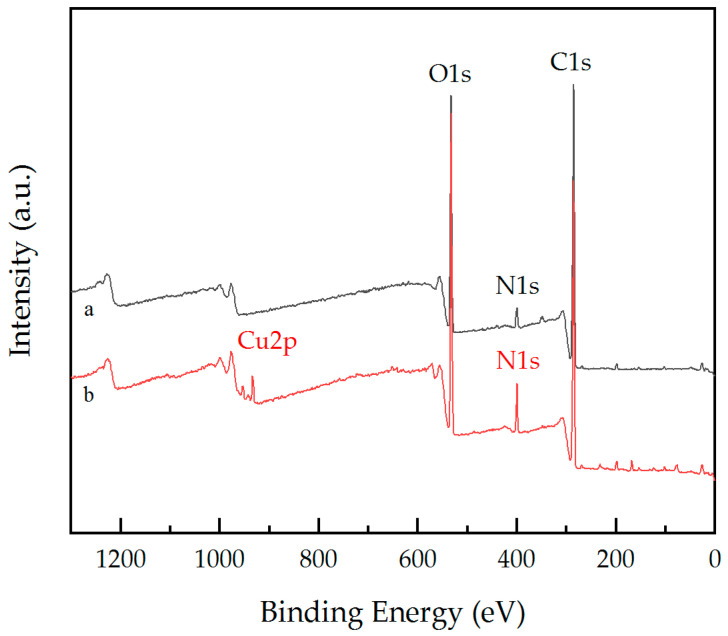
XPS spectra of the as-prepared paper samples before (**a**) and after (**b**) the adsorption of Cu^2+^.

XPS was used to analyze the composition of elements and the interaction between components on the paper samples. As shown in Figure 10, after the adsorption of Cu^2+^, a new peak appears at the binding energy of 970.68 eV, where the peak corresponds to the binding energy of Cu^2+^, indicating that the as-prepared paper samples have an adsorption capacity for Cu^2+^.

**Figure 11:**
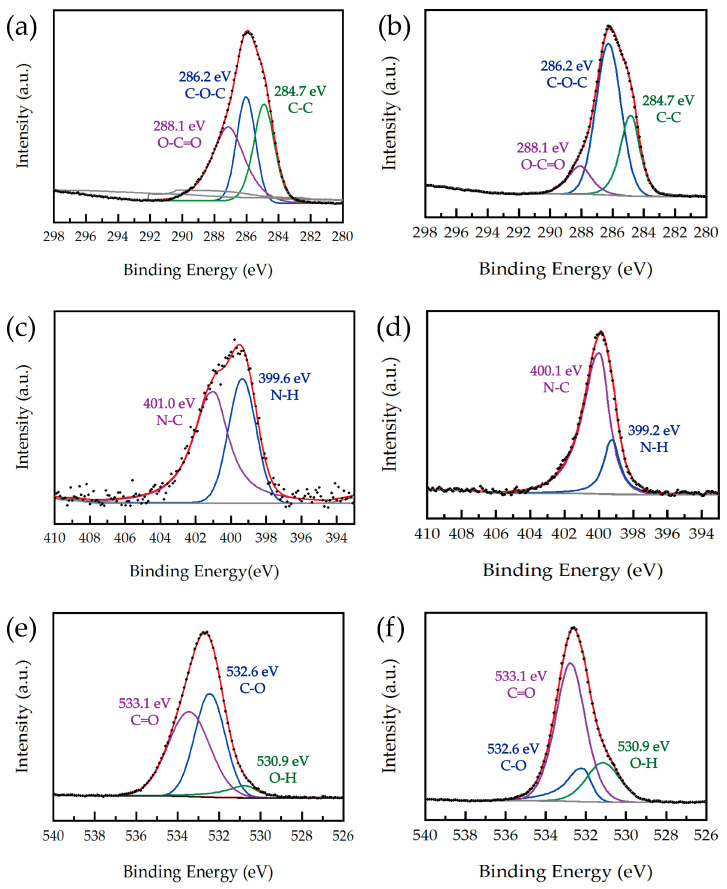
C1s, N1s, and O1s spectra of the as-prepared paper samples before (**a**,**c**,**e**) and after (**b**,**d**,**f**) the adsorption of Cu^2+^.

The chemical environment of the elements was further analyzed according to the high-resolution spectra. For the high-resolution spectrum of N1s (Figure 11c,d), there are two peaks; the binding energy at 399.6 eV before adsorption is attributed to N-H, and the binding energy at 401.0 eV is attributed to N-C. After adsorption, the position and intensity of the above binding energy change, indicating that Cu^2+^ interacts with the amino group of PEI via coordination with a ratio of one to four. However, the binding energy of O1s and C1s did not change in the high-resolution spectra before and after the adsorption of Cu^2+^. The results indicate that the as-prepared paper samples have a good adsorption capacity for Cu^2+^, thereby achieving recognition ability for Cu^2+^.

**Figure 12:**
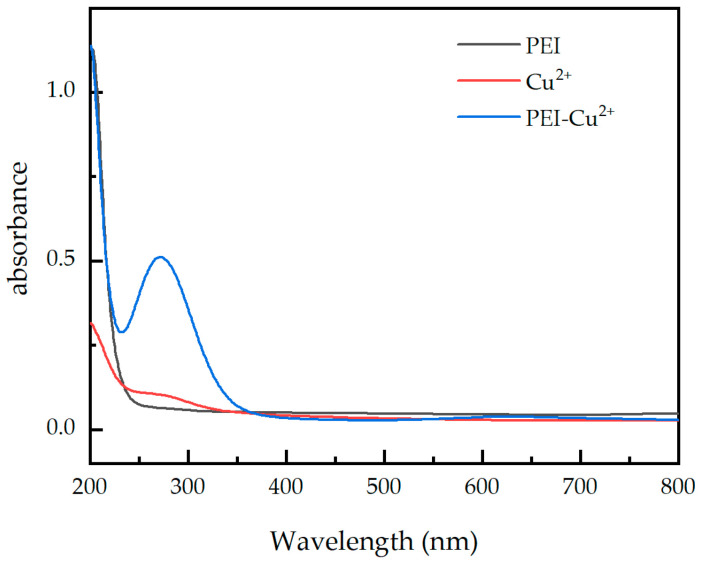
UV–vis absorption spectra of PEI, Cu^2+^, and PEI–Cu^2+^.

The mechanism of fluorescence quenching of Cu^2+^ was investigated using UV–vis spectroscopy. As can be seen from Figure 12, PEI did not show a significant absorption peak in the range of 200~800 nm. However, the solution obtained after mixing PEI with Cu^2+^ has a strong absorption peak at 273 nm, indicating that the PEI–Cu^2+^ complex is formed [24]. The absorption peak of the complex overlaps with the excitation spectrum of the paper-based fluorescence sensors, which weakens the excitation light intensity, and then reduces the fluorescence intensity of the paper-based fluorescence sensor. Therefore, the fluorescence quenching mechanism of the as-prepared paper samples may mainly be due to the internal filtration effect.

### 3.5. Analysis of Actual Water Samples with Paper Samples

Finally, the application potential and accuracy of paper-based fluorescence sensors in actual water samples were evaluated. As shown in Table 1, the relative standard deviation (RSD) of the paper samples for detecting Cu^2+^ in tap water and lake water is in the range of 2.46~3.23%, and the relative error of the BCO method is in the range of 1.95~2.34%. These above results show that the as-prepared paper samples in this work have a high accuracy and sensitivity in the detection of Cu^2+^ in water, and can be used as fluorescent sensors to detect Cu^2+^ in actual water.

## 4. Conclusions

Here, we have prepared a paper-based fluorescence sensor and investigated its potential use in detecting the Cu^2+^ content of water. The paper-based fluorescence materials were prepared by dipping the pristine paper into an aqueous PEI solution based on the aggregation-induced luminescence effect of PEI. The as-prepared paper-based fluorescence sensors can visually and quantitatively detect Cu^2+^ in water based on the inner filtration effect of the PEI–Cu^2+^ complex. The minimum detection limit is 0.03 μM, which is far lower than the maximum permissible concentration in the drinking water, as stipulated by the World Health Organization. When the paper-based fluorescence sensors were applied for the detection of Cu^2+^ in actual water samples, the relative errors were in the range of 1.50~2.30% compared with the BCO method (0.63~630 μM), showing that the as-prepared paper-based fluorescence sensors have a high accuracy and sensitivity, and can meet the requirements for the detection of Cu^2+^ in real water samples. In addition, the paper-based fluorescence sensors are low cost, easy to use, and portable. Therefore, paper-based fluorescence sensors present a good application potential in the detection of Cu^2+^ in water.

## Figures and Tables

**Table 1:** The content in actual water samples.

Samples	Fluorescence Method		BCO Method ^1^		Relative Error (%)
	Observed (μmol/L)	RSD (%)	Observed (μmol/L)	RSD (%)	
**Tap water 1**	4.26	3.12	4.35	2.65	2.11
**Tap water 2**	4.69	3.23	4.80	2.86	2.34
**Tap water 3**	4.11	3.07	4.19	2.76	1.95
**Lake water 1**	5.35	2.76	5.46	2.41	2.06
**Lake water 2**	5.73	2.68	5.85	2.32	2.09
**Lake water 3**	5.58	2.46	5.70	2.11	2.15

^1^ BCO, diacetaldehyde oxaldihydrazone spectrophotometry.

## Data Availability

The original contributions presented in the study are included in the article, further inquiries can be directed to the corresponding authors.

## References

[1] Balasubramanian K. (2016). Quantum chemical insights into Alzheimer’s disease: Curcumin’s chelation with cu(II), Zn(II), and Pd(II) as a mechanism for its prevention. Quantum Chem..

[2] Pal A., Prasad R. (2014). An overview of various mammalian models to study chronic copper intoxication associated Alzheimer’s disease like pathology. Biometals.

[3] Guo Y., Yang L., Li W., Wang X., Shang Y., Li B. (2016). Carbon dots doped with nitrogen and sulfur and loaded with copper(II) as a “turn-on” fluorescent probe for cystein, glutathione and homocysteine. Microchim. Acta.

[4] Zietz B.P., Dieter H.H., Lakomek M., Schneider H., Schneider H., Dunkelberg H. (2003). Epidemiological investigation on chronic copper toxicity to children exposed via the public drinking water supply. Sci. Total Environ..

[5] Barnham K.J., Masters C.L., Bush A.I. (2004). Neurodegenerative diseases and oxidative stress. Nat. Rev. Drug Discover..

[6] Wang Y.H., Zhang C., Chen X.C. (2016). Ratiometric fluorescent paper sensor utilizing hybrid carbon dots-quantum dots for the visual determination of copper ions. Nanoscale.

[7] Chereddy N.R., Janakipriya S., Korrapati P.S., Thennarasu S., Mandal A.B. (2013). Solvent-assisted selective detection of sub-micromolar levels of Cu^2+^ ions in aqueous samples and live-cells. Analyst.

[8] Doner G., Ege A. (2005). Determination of copper, cadmium and lead in seawater and mineral water by flame atomic absorption spectrometry after coprecipitation with aluminum hydroxide. Anal. Chim. Acta.

[9] Pourjavid M.R., Arabieh M., Yousefi S.R., Sehat A.A. (2016). Interference free and fast determination of manganese(II), iron(III) and copper(II) ions in different real samples by flame atomic absorption spectroscopy after column graphene oxide-based solid phase extraction. Microchem. J..

[10] Jiang X., Gan W., Wan L., Deng Y., Yang Q., He Y. (2010). Electrochemical hydride generation atomic fluorescence spectrometry for detection of tin in canned foods using polyaniline-modified lead cathode. J. Hazard. Mater..

[11] Chen L., Lei Z., Hu K., Yang S., Wen X. (2018). Non-aqueous phase hydride generation and determination of trace bismuth by atomic fluorescence spectrometry. Microchem. J..

[12] Becker J.S., Matusch A., Depboylu C., Dobrowolska J., Zoriy M.V. (2007). Quantitative imaging of selenium, copper, and zinc in thin sections of biological tissues (slugs-genus Arion) measured by laser ablation inductively coupled plasma mass spectrometry. Anal. Chem..

[13] Xie Y.L., Zhao S.Q., Ye H.L., Yuan J., Song P., Hu S.-Q. (2015). Graphene/CeO_2_ hybrid materials for the simultaneous electrochemical detection of cadmium (II), lead (II), copper (II), and mercury (II). J. Electroanal. Chem..

[14] Niu L.M., Luo H.Q., Li N.B., Song L. (2007). Electrochemical detection of copper(II) at a gold electrode modified with a self-assembled monolayer of penicillamine. Anal. Chem..

[15] Mi X., Zhang T., Zhang C., Wang Y., Chen H., Li J., Fu Z., Zhang Z., Zheng H. (2021). Plasmonic Sensing of Cu^2+^ via Shell-Etching of Au@Ag Nanorods. Mater. Chem. Phys..

[16] Timur A. (2018). Doped Carbon Dots for Sensing and Bioimaging Applications: A Minireview. Nanomaterials.

[17] Klein G., Kaufmann D., Schürch S., Reymond J.-L. (2001). A fluorescent metal sensor based on macrocyclic chelation. Chem. Commun..

[18] Sun L., Hao D., Shen W., Qian Z., Zhu C. (2012). Highly sensitive fluorescent sensor for copper (II) based on amplified fluorescence quenching of a water-soluble NIR emitting conjugated polymer. Microchim. Acta.

[19] Cano-Raya C., Ramos M.D.F., Vallvey L.F.C. (2005). Fluorescence quenching of the europium tetracycline hydrogen peroxide complex by copper(II) and other metal ions. Appl. Spectrosc..

[20] Leth S., Maltoni S., Simkus R., Mattiasson B., Corbisier P., Klimant I., Wolfbeis O.S., Csöregi E. (2002). Engineered bacteria based biosensors for monitoring bioavailable heavy metals. Electroanal. Int. J. Devoted Fundam. Pract. Asp. Electroanal..

[21] Xing C., Shi Z., Yu M., Wang S. (2008). Cationic conjugated polyelectrolyte-based fluorometric detection of copper(II) ions in aqueous solution. Polymer.

[22] Fan J., Zhang S., Li F., Shi J. (2020). Cellulose-based sensors for metal ions detection. Cellulose.

[23] Li Y., Tang Z., Wang W., Huang X., Lv Y., Qian F., Cheng Y., Wang H. (2021). Improving air barrier, water vapor permeability properties of cellulose paper by layer-by-layer assembly of graphene oxide. Carbohydr. Polym..

[24] Zhao X., Chen H., Wang S., Wu Q., Xia N., Kong F. (2018). Electroless decoration of cellulose paper with nickel nanoparticles: A hybrid carbon fiber for supercapacitors. Mater. Chem. Phys..

[25] Shi F., Cui S., Liu H., Pu S. (2020). A high selective fluorescent sensor for Cu^2+^ in solution and test paper strips. Dye. Pigment..

[26] Sadollahkhani A., Hatamie A., Nur O., Willander M., Zargar B., Kazeminezhad I. (2014). Colorimetric disposable paper coated with ZnO@ ZnS core–shell nanoparticles for detection of copper ions in aqueous solutions. ACS Appl. Mater. Interfaces.

[27] Zhang M., Zhang J., Che X., Jiang J., Tu Q., Wang J. (2024). Biomimetic mineralization-based in situ growth of AuNCs@ ZIF-8 on paper fibers for visual detection of copper ions. Talanta.

[28] Yan X., Zhang L., Zhang Y., Yang G., Yan Z. (2011). Amine-Modified SBA-15: Effect of Pore Structure on the Performance for CO_2_ Capture. Ind. Eng. Chem. Res..

